# National guidelines and good practices on unmet needs in self-care, education, training, and digital tools in person-centered dementia care models across Europe–a cross-country comparative analysis

**DOI:** 10.3389/fmed.2026.1869995

**Published:** 2026-07-22

**Authors:** Nadia Blecha, Anna-Karin Holst Johannsen, Magdalena Kuźma-Kozakiewicz, Pawel Cwiek, Tomasz J. Sarnowski, Lina Danusevičiene, Mariia Rosol, Latchezar Trayko, Shima Mehrabian, Natko Gereš, Karmen Korda Orlović, Zane Anna Litauniece, Nuria Moreno García, Birutė Bartkevičiūtė, Jochen René Thyrian

**Affiliations:** 1 German Center for Neurodegenerative Diseases (DZNE), Greifswald, Germany; 2 Center for Assisted Living Technology, Health and Care Department, Aarhus, Denmark; 3 Department of Neurology, Medical University of Warsaw, Warsaw, Poland; 4 Institute of Biochemistry and Biophysics, Polish Academy of Sciences, Warsaw, Poland; 5 Mental Health Division, Lithuanian University of Health Sciences (LSMU), Kaunas, Lithuania; 6 Public Health Center of the Ministry of Health of Ukraine, Kyiv, Ukraine; 7 Clinic of Neurology, University Hospital Alexandrovska, Sofia, Bulgaria; 8 Clinic of Neurology, UH “Alexandrovska”, Medical University–Sofia, Sofia, Bulgaria; 9 Croatian Institute of Public Health (CIPH), Zagreb, Croatia; 10 Institute of Public Health, Riga East University Hospital, Rīga Stradiņš University, Riga, Latvia; 11 Foundation for the Training and Research of Health Professionals of Extremadura, Extremadura, Spain; 12 Institute for Community Medicine, University Medicine Greifswald, Greifswald, Germany

**Keywords:** best practices, cross-country comparative analysis, dementia, dementia policies, national policies

## Abstract

**Introduction:**

People with dementia and their caregivers regularly face unmet needs, not only in dementia care, but also in self-care, education, training, and the use of digital tools. National dementia policies, clinical guidelines, and best practices can improve dementia care and quality of life across Europe. This study examined how far current national guidelines and good practices address these domains, and where important gaps remain. The objective was to identify unmet needs in national guidelines and good practices on self-care, education and training, and digital tools for people with dementia and their caregivers.

**Methods:**

We conducted a cross-country comparative analysis in countries participating in the JADE Health collaboration: Bulgaria, Croatia, Denmark, Germany, Latvia, Lithuania, Poland, Spain, and Ukraine. Country partners provided national and regional dementia guidelines and descriptions of good practices, which were systematically reviewed and compared against selected best practices from the EU Best Practice Portal, focusing on alignment and gaps in the three domains.

**Results:**

We found substantial variation between countries in the maturity, scope, and implementation of dementia guidelines and good practices. Some countries, such as Denmark and Germany, have relatively advanced policy frameworks and clinical guidance, while others, such as Croatia, rely on promising but regionally limited programs, and several, including Bulgaria and Latvia, still depend on fragmented services. Across all countries, integrated approaches that combine structured self-care support, systematic education and training, and scalable digital tools were rare.

**Discussion:**

Best practices explicitly addressing self-care, education and training, and digital tools remain limited and unevenly implemented. Strengthening and disseminating such practices is essential to guide the further development of dementia care across Europe and to reduce persistent unmet needs among people with dementia and their caregivers.

## 1 Introduction

In the face of an aging population, increasing cases of dementia have become a global challenge (1). By 2050, cases are estimated to surpass 150 million (2), imposing a heavy economic burden on formal and informal care systems (3). Dementia is a multifaceted neurodegenerative disorder that affects memory, cognitive abilities, and behavior (4). In the best-case scenario, its management consists of a multidisciplinary approach, engaging varied healthcare professionals and treatments reflecting its complexity (5). Nevertheless, reality for patients with dementia looks rather different, with delayed diagnoses and lack of transferal to specialists (6), just to name a few. Therefore, it’s not surprising that many people with dementia and their caretakers experience unmet needs in multiple areas (7, 8).

Unmet needs regularly revolve around nursing treatment and care, social counseling and legal support, and pharmacological treatment and care (9), and have been associated with anxiety and depression (10). Additionally, self-care, education, and training have been reported as areas where people affected by dementia experience unmet needs (8, 11). These are not only due to lack in provision of care but also arise from gaps in policy frameworks that shape how dementia care is organized, resourced, and delivered (12).

In dementia care, policy making can play a pivotal role in the delivery of diagnosis, treatment, and ongoing support services. This is achieved through developing national dementia strategies, legislative and regulatory frameworks, clinical guidelines, and accreditation standards (12, 13). These instruments do not only define expectations for care quality, but also outline professional competencies, and set forth the legal and ethical foundations that govern the rights and protections of people living with dementia and their caretakers (14). For example, early detection of dementia is influenced by screening protocols, referral systems, and the distribution of diagnostic resources, which is all determined by said policies (14, 15). Additionally, they guide treatment practices by specifying evidence-based therapeutic approaches and care pathways, thus promoting person-centered care (13, 16). But policies cannot work on their own, they work interdependently with joint actions and best practices.

Best practices can help and ensure a high qualitative standard and help guide practitioners in supporting patients with dementia and their caretakers. To optimize the treatment and care of patients with dementia, several best practices have emerged over the last couple of years. “Best practice” refers to a method or technique that has been proven to produce optimal results, often adapted as a standard for a particular field. To better disseminate already existing best practices, the European Commission has created a database as a repository to assist the identification, collection, and exchange of best practices, namely the EU Best Practice Portal (17). One such initiative that seeks to put these best practices into action is the JADE Health Joint Action (18).

The JADE Health Joint Action (JA) is a collaborative EU initiative with the main goal to decrease the societal and personal load of dementia and other neurological disorders with cognitive impairment through developing new care models, which will promote awareness, healthy aging, and early detection. It integrates best practices and/or interventions by implementing 44 pilots in 17 participating EU countries. The objectives are to ensure best practices chosen lead to sustainability, to develop and encourage new health treatments, to create equitable access to care, and to support new policies. It aims at fighting stigma, especially in vulnerable groups, promoting new person-centered care models, improving the efficiency of promotion and prevention campaigns, and assuring broad dissemination of JADE Health activities and outcomes to enhance health literacy and data accessibility. JADE Health is organized into ten work packages and is implemented by 47 organizations to promote collaboration, shared learning, and long-term sustainability (19). The first four work packages center around management and coordination (WP1), communication and dissemination (WP2), evaluation (WP3), and sustainability (WP 4). Work packages 5 – 10 are focusing on health literacy and data accessibility (WP 5), early detection of neurocognitive disorders (WP 6), harmonized and synergistic prevention of dementia and stroke (WP 7), care pathways for persons with dementia and neurodegenerative disorders (WP 8), person-centered new care models (WP 9), and vulnerable groups and stigma awareness raising (WP 10) (20).

WP 9, of which this analysis is part of, aims to identify gaps in current best practices for emerging European care models – particularly for dementia and other neurological disorders accompanied by cognitive impairment – by reviewing existing approaches, comparing them with guidelines, and improving person-centered care models. Its objectives include enhancing the economic and social sustainability of new care models, helping healthcare professionals improve treatment quality to support patient autonomy and reduce caregiver burden, involving families and stakeholders throughout the project, and integrating aging-in-place principles with new technologies and person-centered care approaches (21).

This cross-country comparative analysis aims at identifying gaps in new care models by summarizing all available best practices on the topics self-care, education and training, and supportive digital tools for patients with dementia and their caretakers. It mainly focuses on describing the current landscape and the status quo and comparing the different developmental stages of specific European countries.

## 2 Methods

### 2.1 Study design

We conducted a cross-country comparative analysis, in which we examined national guidelines and good practices on self-care, education, training, and digital tools in dementia. Additionally, we highlighted recent national and regional developments and programs to give a comprehensive overview. To give a broad overview and to increase comparability, this analysis employs a narrative and descriptive design.

### 2.2 Document identification

For data collection, we contacted team members from all countries involved in the work package, namely Bulgaria, Croatia, Denmark, Germany, Latvia, Lithuania, Poland, Spain, and Ukraine per e-mail, asking them to send us their country-specific guidelines and best practices. The first e-mail was sent out on the 07th of April 2025, setting a deadline for the 30th of April, as discussed in a Teams meeting beforehand. In a meeting on the 16th of April 2025, we discussed the deadline and it was agreed to extend the deadline and move it to the 12th of May. All of the guidelines and practices were received by the 30th of May, only two partners needed to have reminders sent, and only one partner needed multiple reminders. The best practices used for comparison were retrieved from the EU Best Practice Portal. This is a platform created by the EU commission, aiming at addressing public health challenges of Member States by identifying, collecting, and transferring best and promising practices, and to reach the health-related Sustainable Development Goals by 2030 (17). Best practices were retrieved through the best practice search engine on the portal, and filtering for topics of interest, such as “Integration of treatment, management and care” and “Provision of community-based health services.”

### 2.3 Analysis

All documents were systematically compared against a predefined best-practice framework. Analysis focused on identifying areas of alignment, divergence, and gaps. Findings were synthesized narratively and presented using structured tables and matrices to facilitate cross-country comparison.

### 2.4 Best practices

#### 2.4.1 Memory and Cognition Consultation Program

The Memory and Cognition Consultation Program (MCC) (22) involves systematic treatment with a wide range of staff from different specialties, and includes all people involved in treatment of the patient, such as the patient themselves, the caregiver, the family, and primary care. The primary objective is to improve the diagnosis and treatment of dementia, and the quality of life of patients and caregivers.

It aims to foster early diagnosis and treatment to promote quality of life, decrease the number of readmissions, increase timely interventions in ambulatory care, and provide support to the caregiver. Its scope also encompasses promoting knowledge about the disease and its implications, improving coordination between the various services and technical stakeholders in patient care, and increasing the number of patients covered by the MCC. Furthermore, it seeks to reduce hospital costs through coordinated and systematic intervention, increase the level of satisfaction for both the patient and caregiver by improving available information, decrease caregiver burden, and promote the dissemination of the project within the scientific community. This fosters a better characterization and approach to dementia within the MCC in order to facilitate replication in other institutions.

The activities include initial assessment by a multidisciplinary team of specialists, followed by a joint discussion regarding the initial assessment and the patient’s integration criteria in cognitive rehabilitation, psychoeducation, and support groups. During a monthly meeting, diagnostic questions, treatment plan, the need for additional support, and neuro-radiological tests are discussed. When the clinical benefit does not justify maintaining MCC, the patient is discharged and caregivers are referred to a subprogram of nursing consultation.

#### 2.4.2 Smart Aging Mindbrain

The first part of Smart Aging Mindbrain (23), SMARTAGING, exploits the concepts of preventive medicine. It includes instructions for healthy lifestyle, tele-monitoring of daily activities, training of cognitive functions, tele-monitoring of physiological parameters, and automatic feedback about a subject’s response. The second part, MINDBRAIN exploits the use of MRI and EEG biomarkers for an early diagnosis of Alzheimer’s disease (AD).

Mindbrain addresses a multitude of challenges, such as unhealthy lifestyles, especially in patients with chronic comorbidities such as renal diseases. It deals with misleading sources of information about healthy nutrition and physical activity as a function of age and general health status. It manages the need for periodic control of physiological parameters and behavior to verify the adherence to activities in older individuals and people with chronic diseases. It also deals with the lack of quantitative and repeatable measurements of cognitive functions in older people during periodical visits to family doctors. Most of the diagnosis of dementia occur too late and interventions are less effective.

SMARTAGING and MINDBRAIN services offer services for clinical research on the tele-monitoring and conditioning of healthy lifestyle for active aging and early diagnosis of several cognitive disorders thanks to advanced MRI and EEG biomarkers. The innovative elements include an innovative “Information and Communication Technology” (ICT) based tablet battery of 8 cognitive tasks for daily assessment of cognitive functions and brain training, automatic composition of a report on the subjects’ lifestyle and vital and physiological parameters as a feedback for the patient without the involvement of medical doctors in person. Apulian neurological centers are aligned with the most advanced procedures for the extraction of magnetic resonance imaging (MRI) and electroencephalogram (EEG) biomarkers for early diagnosis of AD.

#### 2.4.3 MARIO–managing active and healthy aging with use of caring service robots

MARIO (24) is a European Union-funded project (PHC-19-H2020) using service robots to address loneliness, and isolation in older adults with dementia. It utilizes semantic data analytics, personal interaction, and custom applications to connect older adults with caregivers, community, and personal interests. It focuses on delivering multi-faceted, user-centered interventions through robots in healthcare settings.

This best practice aims at addressing main challenges in caregiving. It aims at tackling complex issues of loneliness, isolation, and early-stage dementia. It supports caregivers and physicians in conducting Comprehensive Geriatric Assessment (CGA). It also deploys flexible, modular, and cost-effective robotic platforms and manages to move robotics from lab settings to real-world healthcare environments. CGA was entirely manual, time-consuming and resource-intensive. MARIO aims to support CGA by autonomously gathering health data via sensors and natural language processing. It is the first robot-based CGA assistant in healthcare practice.

Robots can non-intrusively monitor activities of daily living and cognitive tasks. They help to assess independence in both basic (e.g., bathing, feeding) and advanced activities (e.g., managing finances, medication). Additionally, they aid in cognitive assessment and improve frequency and quality of CGA. No major additional resources are required. Therefore, it supports the redistribution of existing resources across healthcare, social services, and education sectors.

## 3 Results

### 3.1 Bulgaria

Southeast European countries are facing critical gaps in dementia care due to rural-urban disparities, workforce shortages, and limited access to specialized services (25, 26). As a country in Southeast Europe (SEE), Bulgaria is subject to the general regional trends and its characteristic historical and political trajectories. The healthcare system, and in particular care for people with dementia, is directly linked to the country’s demographic and socioeconomic characteristics, the age structure of the population, and economic capabilities.

Although there is no National Dementia Plan, in 2019, due to the efforts of an expert working group appointed by the Ministry of Health and led by the Bulgarian society of dementia, a National Strategy for diagnosis, treatment, prevention, and care of dementia was submitted, and is pending approval by the Council of Ministers. The concept was incorporated into the National Health Strategy until 2030 and adopted by the Ministry of Health in 2020. An action plan has been developed for the National Strategy for the Mental Health of Citizens of the Republic of Bulgaria 2021–2030 (as of November 2021). Programs have been developed for the prevention and early diagnosis of symptoms of cognitive impairment and various forms of dementia (Expert Council on Psychiatry, Ministry of Health, National Center for Public Health and Analyses).

The first memory center in Bulgaria was established in 2001. It was approved by the Ministry of Health as an expert center for diagnosis, treatment, and care of rare neurodegenerative diseases with cognitive, behavioral, and movement impairments in 2023. Seven other memory centers have been established over the past 25 years. There is a revised national consensus on the diagnosis, treatment, and care of dementia in 2015, which was approved by the Ministry of Health. Additionally the role of GPs in dementia detection was well defined, and a toolbox was introduced.

Bulgaria develops and implements European initiatives and programs through the Bulgarian Dementia Society and in partnership with other non-governmental organizations and engaging state institutions. Bulgaria has participated in a regional network focused on promoting innovative interventions, including digital learning formats, caregiver support platforms and region-specific programs, as well as aimed at improving prevention, diagnosis and the long-term management of dementia. The results of a regional study on the key challenges in dementia care across southeastern countries were translated into regional initiatives and projects aimed at addressing gaps through interdisciplinary collaboration, digital innovations, and capacity building (25, 26).

In Bulgaria, through the Danubian Network for Dementia Education and Care (DANDEC), a regionally tailored e-learning program was developed, and addressed unmet needs in dementia care focused on improving professional skills, integrating evidence-based care frameworks, and developing assistive technology strategies. The project emphasized interdisciplinary collaboration through initiatives like the Dementia Academy and introduced the Circle of Care Hub framework to coordinate services among healthcare providers, social workers, and families under the guidance of dementia coordinators. Participating in the INDEED (Innovation for Dementia in the Danube Region) project, launched in 2018, further expanded cross-border collaborations among 11 countries through web-based platform offering multilingual, mobile-accessible content for public awareness, professional training, as well as business development. By addressing infrastructure limitations, INDEED promoted scalable interventions tailored to diverse contexts, including early diagnosis, and multidisciplinary care. Focus was also put on dementia-friendly communities. The development of the “Inter-professional Management on Dementia” workbook and regional training sessions demonstrated the impact of strong organizational collaboration on improving care outcomes (27).

Further educational programs and digital solutions were provided by the ACCESS Dementia platform and expanded these efforts by integrating features like an easy reading language version, simplified content, and sign language tools to improve accessibility for people with cognitive impairments, reading difficulties, or hearing loss. The platform’s community module connected users to organizations, dementia policies, and feedback mechanisms, while targeted workshops addressed the needs of 24-h caregivers. By combining digital flexibility with tailored content, ACCESS Dementia demonstrated the potential of hybrid solutions to address regional disparities and promote sustained learning.

The educational course on clinical neuropsychology/dementia for neurologists and clinical psychologists at Medical University-Sofia has existed since 2008. There is also an educational course on dementia care with nurses at Medical University – Sofia, Alexandrovska University Hospital, Memory center, since March 2019. Since 2001, there have been regular training programs for GPs and specialists in the field of dementia care. In 2023 through a nationwide initiative of Bulgarian Society of Dementia, more than 100 GPs were provided with a training program to screen patients with risk factors and cognitive impairment.

Bulgaria has participated in a previous joint action program where identified best practices were tested in the areas of diagnosis and post-diagnostic support, crisis and care coordination, residential care and dementia-friendly communities. Digital health solutions and telemedicine was implemented in diagnosis and post-diagnosis support, as well as in residential care. The Bulgarian pilot study IMPACT focused on staff training in a TIME-like model at home for older people and people with dementia.

A program for early diagnosis and prevention of cognitive and behavioral impairment was implemented through the support of the European Union – NextGenerationEU program, the National Recovery and Resilience Plan of the Republic of Bulgaria, and the Strategic Research and Innovation Program for Development of the Medical University in Sofia.

On a community level however, there is poor inter-sectorial coordination (e.g., health and social care) for integrated case management. Multidisciplinary teams in diagnosis of dementia are usually available in expert centers and university hospitals. At a national level, there are still challenges with regards to delays in detection and diagnosis, poor health and social coordination, and poorly regulated crisis management.

In terms of dementia care, there is no joint plan across sectors. The only acting process at a national level is the decision of Medical Disability Commission for social services. At national level, after diagnosed with dementia, the patients are referred by GPs to the nationwide Medical Disability Commission. After the commission’s decision, the patients have certain opportunities for care and financial help. They could take advantage of getting access to a caregiver, meals on wheels, and some other social services. At a municipal level, there are different social and care services for people with dementia. In the Sofia municipality, GPS tracking devices are provided for people with cognitive impairment.

In Bulgaria, nursing homes are highly stigmatized and sending a family member to one is seen as abandonment of family members and relatives. These might be one of the reasons, among others, why only 5% of all dementia patients are being taken care in nursing homes. Home care is becoming an issue in Bulgaria as well, since young people are emigrating to Western countries in Europe, leaving a gap in relatives to fill the role as a caretaker. As a consequence, most people with dementia are in home care, but they suffer from loneliness and have to be supported by digital services and surveyed through GPS and home video surveillance systems. Although alternatives for nursing homes exist, nursing homes still play a major role in care provision for the older population. Unfortunately, they are not sufficient in offering exclusive care for people with dementia. This is due to the most pressing issues in nursing homes, such as insufficient funding, limited capacities for patients with dementia, and stigmatization among the population.

In summary, in self-care, education, training, and digital tools for person-centered dementia care, Bulgaria has accumulated experience, including the testing of models for crisis situations and non-pharmacological therapy, training of care home staff, and the use of telemedicine. Notable progress has been made in education, training, and digital tools through the use of the INDEED training platform, featuring updated, innovative, and distance learning.

The dissemination of approaches and practices remains insufficient. It is limited to large university hospitals in major cities, as is inter-institutional coordination. Future efforts should focus on ensuring access to diagnosis and care for disadvantaged groups, the digitization of consultations, post-diagnostic care, and social services.

### 3.2 Croatia

A major development in Croatian dementia care is the Croatian Action Plan for Dementia Care until 2027, developed within the Strategic Framework for Mental Health Development, and is planned to be implemented by 2030. The Action Plan provides a national policy mechanism designed to scale up and systematize previously fragmented regional initiatives. It introduces four strategic pillars: (1) prevention and destigmatization across the life course, (2) early and systematic identification of cognitive impairment through primary care screening and digital self-assessment tools, (3) expansion of evidence-based, personalized psychosocial interventions including new technologies, and (4) development of integrated community-based services through multidisciplinary teams, local dementia-friendly initiatives, and coordinated health-social care pathways.

As in April 2026, Croatia has a few ongoing services and time-limited programs as well as guidelines aiming at dementia care. In this review, several programs will be introduced, namely the PAM Memory Program, the SPAM – EU project “Svi za pamćenje,” and the Improving Dementia Care in the Danube Region Project.

The PAM Memory Program is an ongoing best practice intended for the prevention, treatment, and rehabilitation of people suffering from dementia, their family members, and caregivers. It is organized within the Day Hospital for Dementia Patients of the Psychiatric Hospital “Sveti Ivan” in Zagreb. It is implemented by a therapeutic and rehabilitation team consisting of specialist psychiatrists, consulting internists and neurologists, psychologists, nurses/technicians, social workers, and occupational therapists, and follows a holistic and individualized approach. Pharmacotherapeutics and non-pharmacological methods are used, such as mental fitness improvement exercises (laughter therapy, remembering happy days, communication, writing, reading, encouraging hobbies, helping others), multisensory therapy (music therapy, aromatherapy, phototherapy), and creative workshops (art and literary workshop). Psychosocial interventions are carried out by the method of counseling, educational and supportive groups and self-help groups. The PAM Memory Program continues to operate as a structured day-hospital service embedded within routine institutional care. It represents an active and sustainable example of multidisciplinary, non-pharmacological post-diagnostic support.

The SPAM – EU project “Svi za pamćenje” was implemented in Zagreb from 2020 to 2022 with the goal to expand community social services to assist people with dementia and their families. The SPAM project offered two new services at the St. Ivan Hospital, an afternoon stay from Monday to Saturday, and a mobile team for transportation to their stay and support in everyday activities. Additionally, education for caretakers and organized psychological support is offered as part of the project to prevent burnout in informal caregivers. The project comes with a brochure written by healthcare professionals for patients with dementia and their relatives, especially caretakers. It informs about the importance of informing oneself on dementia and related symptoms, practical recommendations for caretakers, how caretakers can handle memory problems, how to keep a family member to maintain a healthy mind, and how to communicate with a person with dementia. It also gives advice on how to start early, and how to prevent problems and caretaker burnout. Also noteworthy within the SPAM project is the “Communicating with people with Alzheimer’s and other dementias” manual for former caregivers.

The project “Improving Dementia Care in the Danube Region” was a completed policy-oriented intervention that concluded in 2021, aimed to improve the quality of life of people with dementia and their caregivers. The project involved several countries to encourage a faster and more efficient exchange of best practices, to encourage creativity in solving the problems of people with dementia, and to accelerate legal reforms. The project was not of a scientific nature, but rather aimed to implement practical strategies related to people with dementia. The project had three specific objectives. Improving the knowledge and skills of healthcare professionals, improving cooperation and coordination of key stakeholders in the care of people with dementia, and encouraging the development of entrepreneurship with better care and nursing for people with dementia. Croatia participated as an associated partner through the City of Zagreb. Its contribution lies primarily in knowledge transfer, professional education, and policy alignment, rather than in the establishment of permanent care services.

Promising regional initiatives such as the ongoing PAM Memory Program and completed EU-funded projects including SPAM and INDEED illustrate emerging person-centered approaches; however, these remain regionally limited and are only now being addressed at national level through the forthcoming Action Plan for Dementia Care.

The PAM Memory Program involves a multidisciplinary team and offers individualized interventions, such as psychosocial support, creative and multisensory therapies, and counseling for patients and caregivers. The SPAM project adds community-based services, caregiver education, and practical guidance, while the Danube Region project facilitates knowledge exchange and stakeholder coordination. Beyond these programs, Croatia has developed a range of complementary good practices that strengthen the broader ecosystem of dementia care. These include the Croatian Alzheimer Alliance as a national advocacy and coordination platform; recurring professional education forums such as CROCAD and EDUKAL; the expansion of dementia-friendly community initiatives leading local authority formally committed to dementia-friendly policies. Public engagement initiatives such as the “Memory Run” further contribute to destigmatization and community awareness. While these practices do not constitute structured care pathways, they represent important enabling conditions for person-centered models and align closely with EU principles of participation, human rights, and community inclusion. Their systematic integration into national implementation frameworks could significantly enhance sustainability and transferability across regions.

Despite these existing initiatives, Croatia currently lacks a nationwide implementation of multidisciplinary consultation programs, particularly with respect to structured early diagnosis. Supportive digital tools and assistive robots are not present. Furthermore, the existing programs are regionally limited and not implemented at a national scale. Overall, while Croatia has established structures and programs that partially align with best practices, significant gaps remain in standardized, nationwide approaches for self-care, education and training, and digital tools for patients and caregivers.

### 3.3 Denmark

Denmark has the National Dementia Action Plan 2025, with an overall purpose to make Denmark a dementia-friendly society by 2025. The three national goals for dementia efforts toward 2025 are Denmark to have 98 dementia-friendly municipalities, to have more people with dementia to be investigated, so that a minimum of 80% receive a specific diagnosis, and to reduce the consumption of antipsychotic medication among people with dementia by 50% through improved care and treatment efforts. The dementia action plan contains a total of 23 initiatives divided into five focus areas. The Danish Health Authority is responsible for 11 of the initiatives within all five focus areas. Within focus area 1, early detection and better quality in assessment and treatment, the Danish Health Authority is responsible for initiative 3, 4, 5, and 6. Initiative 3 aims to reduce interdisciplinary assessment and treatment units. It gives recommendations on how dementia assessment can be gathered in a smaller number of interdisciplinary assessment and treatment units, and where geographical spread is prioritized. Additionally, it helps to ensure that people being assessed for possible dementia receive a high and more uniform quality of assessment and treatment. This will be achieved by centralizing dementia assessment and treatment in fewer, interdisciplinary units. Initiative 4 aims at creating new national clinical guidelines in the area of dementia. So far, three guidelines have already been published, namely “dementia and medicine” (2018), “diagnosis of mild cognitive impairment and dementia” (2018), and “prevention and treatment of behavioral and psychological symptoms in people with dementia” (2019). The fourth guideline, “prevention and treatment of behavioral and psychological symptoms in dementia,” is expected to be published in 2025. Initiative 5 aims at reducing the use of antipsychotics among people with dementia and initiative 6 gives national recommendations for optimal cross-sectoral and interdisciplinary courses. Focus area 2 aims at better quality in care and rehabilitation. Initiative 7 aims at distributing handbooks with knowledge-based recommendations for social and health care practice in the area of dementia, and initiative 8 aims at increasing and improving offers of physical training and activity. Focus area 3 aims at supporting people with dementia and their relatives. Initiative 12 aims at creating more and more meaningful day and respite services and support for young people with dementia. Initiative 13 aims at creating counseling and activity centers for people with dementia and their relatives, including young people with dementia. Focus area 4 concentrates on creating dementia-friendly housing and a dementia-friendly society through initiative 14, local and nationwide activities to support a dementia-friendly society. Focus area 5, finally, tries to increase level of knowledge and competence. Initiative 19 tries to create a new national research strategy in the field of dementia and research, and initiative 22 practice-oriented competence enhancement in municipalities and regions.

Multiple clinical guidelines and recommendations have been issued by the health authority. Topics covered are the prevention and treatment of behavioral and psychological symptoms of dementia (BPSD), the use of antipsychotic medications, and the organization of diagnostic and treatment units.

Additionally, the National Dementia Research Center has localized a UK guideline focusing on non-pharmacological interventions for BPSD for healthcare professionals and has enforced a 2-years project to create dementia-friendly hospital environments through staff training, workflow optimization, and environmental modifications.

Besides these national actions, other actions on municipal level exist. Noteworthy is a comprehensive collaboration between the Aarhus Municipality and the SOSU Østjylland, which is an education institution for health and care workers. It builds on the pilot project at Skovvang Nursing Home. The aim of this collaboration is to increase person-centered care within its medical and social care provisions and to train 2000 leaders, nurses, therapists, social and health assistants, and support staff from 2023 to 2026. Training involves to increase quality of care in dementia care.

The LIVSTEGN project, led by Aarhus University and VIVE, developed ethical frameworks for using surveillance technologies like GPS and motion sensors in dementia care. A set of guidelines are being implemented in Aarhus Municipality’s nursing homes. Key recommendations include appointing key staff members, specific employees should monitor errors and challenges. Establishing clear responsibility, define who is accountable for ensuring the technologies function properly. Reducing alarm overload, ensure alarms match actual resident needs to prevent unnecessary alerts. Monitoring and adjusting usage, regularly assess technology effectiveness and adapt to changing requirements.

Assistive technology for people with dementia (ReACT) is an app designed to support independence and daily living in people with dementia by addressing cognitive and communicative challenges. The app was initiated in 2015 and follow-up data was collected until 2022. ReACT addressed user-involvement, implementation and research methodology when designing assistive technology for people with dementia. The results underlined the benefits of involving end-users in the design and test of assistive technology, to meet their needs and capacity. Results also showed that uptake and adoption of technology can be promoted by implementation methods tailored to people with dementia and their family caregivers.

Aarhus Municipality has initiated a comprehensive collaboration with SOSU Østjylland (education institution for health and care workers) to promote education in the method of person centered care of all health and care professionals in Aarhus Municipality. To enhance person-centered care across its health and social care services, it aims to train approximately 2,000 employees between 2023 and 2026, including leaders, nurses, therapists, social and health assistants, and support staff. Mainly, the focus is on equipping staff with concrete tools to better understand and respond to the individual needs of citizens, particularly those with dementia. A key component is the dementia simulator, which allows participants to experience cognitive impairments. It builds upon a successful pilot project at Skovvang Nursing Home, where the implementation of person-centered care led to increased calmness and security among residents, a significant reduction in the use of antipsychotic medications, improved staff collaboration and job satisfaction, and enhanced involvement of relatives in care planning.

Nursing homes in Aarhus Municipality use various technologies and assistive devices. The list below is not exhaustive but provides an overall picture: GPS for locating PwD, fall alarm, alarm transmitter, door alarm, and motion sensor.

Denmark has an advanced framework for dementia care, including the National Dementia Action Plan 2025, multiple clinical guidelines, and municipal-level initiatives. The country has established interdisciplinary assessment and treatment units, published national guidelines on dementia diagnosis and treatment, implemented non-pharmacological interventions for BPSD, and developed person-centered care training programs for healthcare staff. Assistive technologies such as GPS tracking, fall alarms, and motion sensors are in use, and apps like ReACT support independence and daily living for people with dementia. Despite these strengths, we were not able to find a best practice or structured multidisciplinary consultation program on digital tools, which doesn’t mean that they do not exist. Overall, Denmark demonstrates a mature and comprehensive dementia care system with partial adoption of digital tools, education/training, and self-care support, but gaps remain in fully integrating the elements exemplified in the selected EU best practices.

### 3.4 Germany

Germany has created the S3-guidelines for dementia from the German society for psychiatry, psychotherapy, psychosomatics, and neurology e.V. (DGPPN). It is an evidence and consent-based guideline for diagnostic, therapy, and prevention of dementia. The guideline is only written for dementia, mild cognitive impairment with Alzheimer’s dementia, mixed dementia, frontotemporal dementia, dementia in Parkinson, and Lewy body dementia. It only includes symptoms directly related to dementia, including psychological and behavioral symptoms. The aim of this guideline is to give support in decision-making to professionals involved in dementia care. The main focus is basic medical area, and the foundation of this guideline are evidence-based medicine and structured consensus finding. It includes state-of-the-art standard for diagnostic, therapy, care management, and guidance to ensure high quality and gives recommendation for or against procedures. The guideline is written for all healthcare areas and for following professions: M.D.s related to dementia care, (neuro)psychologists, ergo therapists, physiotherapists, art therapists, speech therapists, nurses, and social workers. Guidelines exist for consent of diagnostic and treatment, diagnostics, therapy, geriatric care, palliative care, and prevention of dementia and mild cognitive impairment. Guidelines for therapy focus on recommendations for or against dementia care management, cognitive training for dementia, cognitive stimulation, computer-based cognitive therapy outside of professional settings, cognitive procedures in MCI, music therapy, physical training, physical training for improvement of daily activities.

Additionally to this guideline, an expert standard “Relationship-Centered Care in the Nursing of People with Dementia” exist, which was released in January 2018 by the German Network for Quality Development in Nursing (DNQP). The aim of the expert standard was to shift care practices from a purely functional focus toward a person-centered, relationship-oriented approach and therefore, to ensure that every person with dementia receives care that fosters feelings of being heard, understood, accepted, and connected. This standard had been developed from a synthesis of Tom Kitwood’s person-centered care model, international and national literature on dementia care, particularly communication and interaction interventions and expert consensus. The standard is organized into structure, process, and outcome criteria. Structure data was centered around person-centered attitude (S1a), competencies to identify relational support needs (S1b), and institutional fostering and supportive organizational environment (S1c). Process criteria involve comprehensive assessments of relational support needs, forming empathy toward the individual’s experience and behavior (P1), planning and coordinating customized relational care (P2), and informing, guiding, and advising the person with dementia and their relatives about relational interventions (P3). Outcome criteria include recognition of individual’s uniqueness (E1a), detailed documentation of relational needs (E1b), planning of tailored relational measures known to all caregivers (E2), execution of relationally supportive care (E4), and ongoing evaluation capabilities (S5a).

The model-based implementation of the expert standard “Relationship-Centered Care in Dementia” was designed to evaluate its practicality and acceptance across diverse care settings. From January to June 2018, for 6 months was conducted in 29 institutions. A four-phase model guided the process. Staff was educated on the standard, the standard was adapted concretely to each setting’s specifics, the supervised trial was guided using the standard in practice, and the implementation being evaluated via internal audits. They found a general practicability and acceptance across different care settings and the structured, phase-based methodology (preparation, training, customization, introduction, and audit) to be effective.

Noteworthy, though not a guideline or good practice, are the memory clinics across Germany, which are affiliated with larger hospitals and university hospitals. They are drop-in-centers for people concerned about their memory and offer early diagnosis and guidance.

Germany has developed comprehensive clinical guidelines (S3-guidelines) and an expert standard for relationship-centered care, covering diagnostic, therapeutic, and preventive aspects of dementia, and providing support for a range of healthcare professionals. The S3-guidelines address cognitive training, physical activity, and psychosocial interventions, while the expert standard emphasizes person-centered, relational care with structured implementation and evaluation. Memory clinics across Germany offer early diagnosis and guidance, though regulation and standardization vary. Despite these strengths, Germany currently lacks a structured multidisciplinary consultation program. Education and training for patients and informal caregivers, as a structured, standardized element, are not covered in existing guidelines or good practices. Supportive digital tools and assistive robots are absent. Overall, Germany has mature medical and relational care frameworks, but gaps remain in integrating the full scope of best practices in self-care, education/training, and digital tools at a standardized, national level.

### 3.5 Latvia

At the end of 2016, Latvia had a prevalence of only 0.3%, suggesting that dementia is not being effectively diagnosed and treated in Latvia. Additionally, the fact that Alzheimer’s dementia is not the most commonly diagnosed type of dementia in Latvia raises concerns about the accuracy of diagnostics in Latvia. A clinical pathway, “Dementia Assessment and Diagnosis,” exists. The objectives of the Clinical Pathway are to improve early detection at the primary care level, to promote more accurate differentiation of dementia types, to use diagnostic tools rationally and ensure purposeful referrals to specialists, and to reduce inefficient use of healthcare resources and optimize diagnostic planning for patients.

According to the clinical pathway, initial assessment should be done by the GP. The GP gathers information from the patient and their close contacts on memory difficulties, changes in behavior, emotional regulation difficulties, daily life difficulties (information from both patient and close person). They evaluate the patient’s general health and exclude other reversible causes of cognitive impairment (urine analysis for infections, medication, pain and trauma history, nutritional and vitamin deficiency, alcohol abuse, electrical imbalance, visual or hearing impairments, delirium, depression). The GP diagnoses a dementia syndrome, using either the Montreal Cognitive Assessment, the MMSE, or a combination of the 5-Word Recall Test from the MoCA, the clock drawing test, and the orientation assessment. They involve patients and their relatives in decision-making and provide information, e.g., respect for patient, thorough information for patient and relatives. In cases of mild dementia, the general practitioner (GP) refers for a brain magnetic resonance imaging (MRI) to rule out other potentially surgically treatable pathologies. The GP then refers to a neurologist or psychiatrist if all of the following three criteria are met: other reversible causes have been ruled out, persistent signs of dementia, and patient has mild dementia. The GP refers the patient to a specialized neurology day hospital or a geronto-psychiatric clinic (does currently not exist in the publicly funded system) if all of the following 3 criteria are met: other reversible causes have been ruled out, persistent signs of dementia, and patient has moderate to severe dementia.

In secondary care stage, following specialists can be involved: a specialized neurology day hospital or gerontopsychiatric clinic (currently, these medical institutions have not been established and are not included in the state-funded healthcare system), and for cases of moderate and severe dementia: neurologist, psychiatrist, gerontologist (available if necessary), clinical psychologist (available if necessary), social worker, occupational therapist, psychiatric nurse, art therapists (available if necessary), physiotherapist (available if necessary), internist or cardiologist (available if necessary), outpatient visit to a psychiatrist or neurologist in cases of mild dementia. The pathway further specifies, how Alzheimer’s dementia, vascular dementia, Lewy Body dementia, and frontotemporal dementia should be diagnosed.

Latvia has established a clinical pathway for dementia assessment and diagnosis, which outlines procedures for early detection, differentiation of dementia types, and appropriate referrals from general practitioners to specialists. Initial assessments include cognitive testing, evaluation of general health, and involvement of patients and relatives in decision-making. However, secondary care structures such as specialized neurology day hospitals or gerontopsychiatric clinics are not yet established or publicly funded, limiting access to multidisciplinary teams and comprehensive care. Compared to the selected best practices, Latvia currently lacks a structured multidisciplinary consultation program. Education and training for patients and caregivers, supportive digital tools and assistive robots are absent. Overall, Latvia’s dementia care is heavily reliant on primary care, with minimal specialized or integrated services, highlighting substantial gaps across self-care, education/training, and digital support relative to the selected EU best practices.

### 3.6 Lithuania

Lithuania is among the most rapidly aging countries in the European Union. The share of the population aged 65 years and over is projected to increase from 20% in 2019 to 32% by 2050, while the proportion of aged 85 years expected to double well above the OECD average (28). The country faces growing burden of dementia care, with approximately 41,500 people currently living with the condition. This demographic shift places increasing pressure on the health and long-term care systems and requires considerable adaptation of both health and social care frameworks, not only service expansion but also effective guidance, education, and support for people living with dementia and their caretakers.

Despite neurodegenerative diseases being among the leading causes of disability in older age, Lithuania lacks a national dementia strategy or dedicated clinical guidelines. Dementia care is embedded within the broader systems for people with disabilities or chronic illnesses under shared responsibility of the Ministry of Health and the Ministry of Social Security and Labor. As a result, services remain fragmented and unevenly developed with variation eligibility of services across municipalities. In this context, people-centered informational guidance, self-care support and educational resources play an increasingly important role in addressing gaps in service provision and supporting individuals and families to navigate a complex care system with weakly integrated pathways.

Although long-term care has been one of the fastest-evolving fields in recent decades, promoting deinstitutionalization, community-based care, and better integration of health and social services through piloting integrated care provision (29), the formal long-term care sector remains insufficient and requires further strengthening. Workforce capacity remains limited, with only one long-term care worker per 100 people aged 65 years and over, compared with an EU average of four, and around 40% of older people report unmet care needs.

Nearly 90% of older people rely primarily on informal family care, and fewer than 5% receive formal care services, particularly in the early and middle stages of dementia. The formal home-based support remains insufficient at advanced stages of dementia and largely absent in the early stages, when timely intervention could most effectively delay functional decline. This service gap highlights the importance of post-diagnostic support, caretakers education, and tools that promote self-management and functional independence.

Care pathways typically begin within the health-care system, with diagnosis serving as the entry to long-term care services and financial supports. However, diagnostic pathways remain fragmented and largely medicalized, despite the multiple cognitive screening tools and multidisciplinary teams. Family physicians are expected to play a more substantial role in early detection and follow-up, yet in practice patients are frequently referred to psychiatric specialists for diagnostic confirmation, contributing to a bureaucratic and prolonged diagnostic process that delays access to appropriate care (30). Although primary health care centers have the structural capacity to involve case managers, community nurses, occupational therapists and social workers, these professionals are not systematically integrated in post-diagnostic dementia care. Access remains firmly anchored to physician-issued referrals, which are required not only for specialists, health care and nursing services, but also for functionally oriented supports such as assistive technologies or transport services, placing unnecessary pressure on physicians, limiting the contribution non-medical professionals, and reinforces reliance on caretakers.

In parallel, access to long-term care supports is organized through Agency for the Protection of the Rights of Persons with Disabilities, typically following referral by a family physician. The Agency conducts needs-based assessments and assigns disability or participation status, on the basis of which individually tailored long-term care plans may be developed, including assistive technologies and financial supports. Eligibility social care services–including day social care, respite care, short-term residential care, and long-term residential care– is determined by municipalities based on needs assessment conducted by municipal social workers, resulting in regional variation in access and service provision.

Ambulation prevention and safety-creating technologies are predominantly oriented for fall prevention in institutional care settings, supported by methodological guidance and external monitoring form the Hygiene Institute (31). In contrast, no specific regulatory requirements, systematic monitoring, or mandated safety technologies exists for home nursing services delivered in the private homes of people living with dementia, revealing a substantial regulatory and safety gap between institutional and home-care settings. In parallel, civil society organizations play an important role in addressing unmet needs in dementia care through advocacy, caregiver education, and accessible, people-centered informational resources. They contribute development of informational and digital resources, such as consolidated guidance on dementia care pathways issued in 2025 by Demencija Lietuvoje (32). However, self-help and peer-support groups for family caretakers are rarely formally recognized or funded as services and rely largely on voluntary engagements, often by social workers, and often dissolve once this support ceases. Dementia-related citizen initiatives are concentrated in the largest urban areas - Vilnius, Kaunas, and Klaipėda - limiting access to such support in smaller municipalities and rural areas. Collectively, these initiatives function as complementary sources of self-care support, and practical guidance, partially compensating for gaps in formal services while also highlighting territorial inequalities in access to dementia-related support.

Despite ongoing systemic efforts aimed at strengthening community based long-term care infrastructure, improving the integration of health and social services, dementia-specific care remains limited in the absence of a comprehensive national dementia strategy. Dementia care continues to be embedded within general elder care frameworks, with notable gaps in self-care support, education and training, and digital solutions relative to EU best practices. Safety and assistive technologies are primarily institution-focused, with no systematic implementation in home-based care. In comparison with selected best practices, Lithuania lacks structured multidisciplinary consultation models, standardized education and training programs for people living with dementia and their caretakers, and the systematic use of digital tools or assistive technologies, including emerging solutions such as assistive robotics. In this context, non-clinical interventions–particularly self-care support, education, training, and digital tools–have the potential to address gaps in service provision, strengthen support for informal caretakers, and improve continuity of care across fragmented pathways. This study therefore examines national guidelines and identified good practices with the aim of mapping existing provisions and to identify opportunities for more coordinated and person-centered dementia care.

### 3.7 Poland

Poland had made several attempts in years between 2011 and 2025 (e.g., 2011, 2014 and 2018) to improve care of patients suffering from dementia, however they were mainly unsuccessful as they represented mostly within societal or organizational initiatives. On the other hand they all lead to the inclusion of some broad recommendations for older people suffering from dementia-related diseases into the general document - the National Health Program for Poland (NPZ, 2021–2025), however there was no strict focus on dementia in this document. Therefore, in the end, Poland issued the National Plan for Dementia Care in Poland (2025–2030) at the end of December 2025. It constitutes the first comprehensive public policy framework addressing the growing burden of dementia in the context of rapid population aging. It concerns dementia resulting from progressive neurodegenerative and vascular conditions with Alzheimer’s disease identified as the most common cause.

Demographic trends in Poland indicate a significant rise in the proportion of older adults, particularly those aged 80 and above, which directly contributes to the increasing number of dementia cases. Current estimates suggest that 370 - 460,000 of individuals are affected, with projections indicating substantial growth by 2040. Dementia already accounts for a notable share of mortality and healthcare utilization, and its impact is expected to intensify in the coming decades. Importantly, a large proportion of cases may remain undiagnosed, particularly in early stages.

The document identifies several systemic challenges. These include insufficient public awareness and persistent social stigma, leading to delayed diagnosis and social exclusion of affected individuals. A critical issue is the heavy reliance on informal caregivers–primarily family members–who experience significant psychological, physical, and financial burdens, often resulting in reduced labor market participation and adverse health outcomes. Additionally, existing support systems are fragmented, lacking coordination between healthcare and social services, and are not adequately tailored to the specific needs of dementia patients and their caregivers.

The overarching objective of the Plan is to increase early detection of cognitive impairment within primary healthcare and to enhance access to comprehensive support for both patients and caregivers. To achieve this, the policy outlines a multi-dimensional approach structured around several key areas. First, the Plan emphasizes the importance of increasing public awareness through educational campaigns, integration of dementia-related content into formal education, and targeted training for non-medical professionals. These measures aim to reduce stigma, improve recognition of early symptoms, and foster supportive social attitudes. Second, the Plan promotes risk reduction strategies based on evidence that a substantial proportion of dementia risk is associated with modifiable factors. These include lifestyle and health-related determinants such as physical inactivity, smoking, cardiovascular risk factors, depression, social isolation, and sensory impairments. Preventive interventions are intended to be implemented across the life course. Third, improving early diagnosis is a priority, particularly through strengthening the role of primary healthcare in screening and referral processes. Earlier identification of cognitive decline is expected to facilitate timely intervention and better care planning. Fourth, the Plan addresses the organization of treatment, care, and support services. It calls for improved coordination between healthcare and social care systems, expansion of community-based services, and enhancement of long-term care provision. Particular attention is given to adapting services to the progressive nature of dementia. Fifth, support for informal caregivers is recognized as essential. Proposed measures include access to psychological support, training, informational resources, and respite services, aimed at mitigating caregiver burden and improving overall care quality. Additional components of the Plan include the development of data collection systems to support evidence-based policymaking and the promotion of scientific research to advance knowledge and innovation in dementia care. In conclusion, the National Plan represents a foundational step toward a coordinated and systemic response to dementia in Poland. By integrating prevention, early diagnosis, care provision, and social support, it seeks to improve quality of life for affected individuals and their families while addressing broader public health and socio-economic challenges.

Importantly, the Plan is broader than a general awareness and caregiver-guidance document, as it is explicitly structured around seven intervention areas: public awareness, risk reduction, early diagnosis, treatment and care, support for informal caregivers, data collection systems, and research.

The program also includes concrete implementation instruments. In early diagnosis, it foresees educational activities for primary care physicians and nurses on the administration and interpretation of cognitive screening tools, as well as the implementation of recommended pathways for preliminary dementia diagnostics in primary care. In caregiver support, it includes the adaptation and dissemination of the WHO iSupport for Dementia educational tool, the operation of a telephone helpline on dementia that also offers psychological support, and pilot psychosocial group support for informal caregivers.

Another important feature is the program’s explicit orientation toward community-based and de-institutionalized care. The document states that hospitalization of people with dementia should be limited whenever possible and that support should be shifted toward care in the home environment, including pilot day-care solutions and education for formal and informal caregivers. In comparative terms, this brings Poland closer to countries that already emphasize community-based support, although implementation mechanisms are only beginning to be operationalized. Besides, there are a number of practical guidelines published at the websites of the Ministry of Health or/and the National Health Fund. Among these there is a guideline for caretakers, with supportive information on how to take care of a dementia patient. It was prepared by the Polish Association Helping People with AD in cooperation with the National Health Fund. It gives advice on how to plan daily activities, ensure a safe home and not lose oneself in the role of a caregiver. It also highlights the important role as a caretaker in the life of a dementia patient. It advices caretakers on how to plan time for an ill person. Examples of activities for the patient include coloring books, puzzles, playing cards, viewing family photos, organizing drawers, and wool rolling. It advices also on how to ensure a safe home. To provide the patient with as much freedom and safety as possible, it is worth adapting the home space. It also advices on how to react to aggressive behavior of the client. The most common causes of aggression in dementia patients are described as disturbances in the daily schedule, attempts to force them to do certain activities, e.g., to bathe, walk, requiring the patient to perform activities that he or she can no longer do, the need to make a choice, and fatigue, pain, discomfort. Finally, the website also gives advice on how to take care of oneself as a caretaker. A caregiver of an elderly person, especially a dependent person, should take care not only of the patient, but also of themselves. Caregiver fatigue can lead to depression, deterioration of health, and a sense of helplessness. Some of the advice given on how to reduce fatigue are: Do less – at least once a week, do less than usual, e.g., prepare sandwiches instead of a two-course dinner. Ask for help – turn to family, neighbors, friends. Let them spend a few hours with the sick person. During this time, do something for yourself. Relax. Use your free time for yourself – if the person you care for uses a day care home, focus on your needs during this time. Synchronize sleep – try to sleep at the time when your pet sleeps.

The National Health Fund has also published guidelines in cooperation with the Ministry of Health and the Ministry of Family and Social Politics for older citizens on receiving external care to support day-to-day activities. It informs on opening hours for primary care facilities and emergency assistance, areas where a GP can be of support, what primary nurses can do, and how to get support. Additionally, it informs on geriatric treatment, long term nursing care at home, and care and treatment facility and nursing and care facility itself.

These practical materials also show that Polish support is not limited to general awareness-raising but already contains relatively detailed post-diagnostic guidance for families. The caregiver guide describes a pathway in which diagnosis usually begins in primary care and may proceed through neurological, psychiatric, neuropsychological, and neuroimaging assessment, while stressing that earlier recognition allows the person with dementia to remain active in family and social life for longer. In addition, it addresses issues that are often overlooked in national comparisons, including legal planning in the early stage of illness, such as notarial power of attorney, later guardianship arrangements, and the organization of daily care. The guidance also covers communication, hygiene, sleep, home safety, behavioral symptoms such as aggression, planning meaningful daily activities, and the need to protect the caregiver’s own physical and mental health through respite, help-seeking, and self-care. This gives the Polish section a stronger practical and person-centered dimension.

At the same time, the report prepared with the participation of the Ombudsman man and Alzheimer organizations indicates that, before the adoption of the 2025–2030 national program, much of the most concrete expertise in Poland had been generated by civil society, clinicians, and local initiatives rather than by a unified state system. These materials repeatedly emphasize delayed recognition, limited epidemiological data, insufficient training of physicians, and weak communication with families, while arguing for earlier case finding in primary care, the use of simple cognitive screening tools, stronger involvement of caregivers in the diagnostic interview, and more structured multidisciplinary pathways linking the general practitioner with neurologists, psychiatrists, geriatricians, psychologists, and social support. They also show that Poland already has a relatively broad advocacy and support network: more than 30 Alzheimer organizations have been described as active in public education, support groups, self-help, and local service development, with some also running day-care or specialized care facilities. In comparative terms, Poland therefore appears to have entered the national strategy phase with a substantial pre-existing advocacy and knowledge base, but with implementation of coordinated multidisciplinary care still uneven and dependent on regional and non-governmental capacity.

Taken together, Poland should no longer be classified as a country without a national dementia strategy. Rather, it should be considered as a country that has recently established a formal national policy framework but is still at an intermediate stage of implementation. Compared with the selected best practices, structured multidisciplinary consultation services and advanced digital tools remain limited, yet the newly adopted program substantially strengthens the policy architecture for self-care, education, training, and caregiver support.

### 3.8 Spain

In 2019, Spain has announced its adaptation of a national dementia strategy, which follows the conclusion of the Neurodegenerative Disease Strategy, which ran between 2016 and 2019, and the WHO Global Action Plan on the Public Health Response to Dementia 2017 – 2025. The national strategy also refers to several European-level initiatives and programs. Additionally, guidelines and informational leaflets for caregivers have been developed as support tools.

The leaflet “*Care for patients with dementia*” provides advice for informal caregivers. It offers guidance on managing memory loss, anxiety, irritability and aggressive behavior, catastrophic reactions, apathy, social withdrawal, wandering, false beliefs (delusions and hallucinations), incontinence, and inappropriate sexual behavior. Its goal is to help the person with dementia maintain the maximum number of tasks possible that can be carried out, without causing frustration for the patient, but help them maintain their dignity as an individual and motivation as a patient. The leaflet also provides recommendations on supporting independence and dignity in daily activities such as household tasks, mealtimes and meal preparation, personal care, and exercise.

It encourages activities with the person with dementia and makes recommendations for creative, intellectual, psychological, social, and spiritual activities, such as painting, doing puzzles, looking at old photographs, having coffee, and reading the Bible. It also lists factors to consider, such as ensuring a safe environment, assigning meaningful tasks to help the person feel useful, establishing a simple routine, maintaining connections with the past, recognizing retained abilities, and identifying preferred or distressing activities, optimal timing for tasks, and potential physical limitations affecting participation.

The guideline “*Advanced dementia, the great challenge*” focuses on communication with relatives in the advanced stages of dementia. It recommends approaching the person slowly from the front, using simple language, and ignoring harmless hallucinations or delusions that do not distress the patient. It also provides practical advice to improve quality of life, such as managing pain, ensuring daily hygiene, regularly changing positions to prevent pressure ulcers, and providing easy-to-swallow food with adequate hydration.

Furthermore, it offers guidance for caregivers on maintaining empathy, patience, and respect, and encourages them to seek help when feeling overwhelmed, share responsibilities, and take time for self-care. The guideline includes self-care, care, and crisis-management strategies. Self-care strategies involve becoming aware of the impact of caregiving, self-assessment, and recognizing warning signs of stress. Care strategies include guidance on mobility and transfers, dressing and footwear, feeding, and elimination. Strategies for dealing with difficult situations focus on problem definition, goal setting, and action planning.

Cuidopía is a Corporate Social Responsibility program by Johnson & Johnson companies in Spain. It aims to highlight the work of thousands of people in our country who, whether formally recognized or not, are dedicated to caregiving in all areas. The program also supports the training of professionals in the field of care through a scholarship program in collaboration with Fundación Tomillo, a social organization specialized in employment training for vulnerable individuals, and SUPERCUIDADORES, an initiative promoted by the International University of La Rioja (UNIR).

The purpose of Cuidopía is to inform, guide, and educate in order to promote a more active, aware, responsible, and care-oriented society. The program also contributes to the United Nations Sustainable Development Goals (SDGs) for 2030. Through guides developed exclusively for the program, it aims to facilitate access for families to basic information on benefits and services under the Dependency Law, including catalogs of services, benefits, and respite programs offered by each Autonomous Community, as well as a consolidated directory of relevant public resources. In addition, www.cuidopia.es provides various caregiving resources and encourages participation and community engagement through social media.

Building on these efforts, Spain has also developed a national strategy to implement a new community-based care model grounded in deinstitutionalization. While this strategy is not exclusively focused on people with dementia, it targets older adults in situations of dependency. Its aim is to transform the system of supports and care so that all individuals–especially those with higher support needs or complex situations–can pursue their chosen life projects within the community, ensuring equal opportunities, autonomy, and quality of life.

This approach recognizes that well-being depends not only on healthcare or social services, but also on social inclusion, active participation, and respect for individual preferences and rights, ensuring that people can live in conditions adapted to their needs rather than being forced to conform to a specific institutional lifestyle.

At the regional level, the region of Extremadura provides a concrete example of implementing person-centered care principles. The Regional Strategic Plan, called “*Untying Knots for Good Treatment*,” targets people with severe and high dependency, as well as individuals with dementia of different types and origins. Developed by the Junta de Extremadura and the Extremaduran Service for the Promotion of Personal Autonomy and Care for Dependency (SEPAD), the plan is a forward-looking strategy adapted to the new realities of residential care.

Its primary goal is to transform the traditional care model into one genuinely centered on the person, moving away from paternalistic or strictly institutional practices and giving the individual a central role in planning and decision-making regarding their own care and life projects. A key aspect of the plan is its commitment to eliminating the use of physical and chemical restraints in residential centers, a measure that both dignifies care and improves residents’ quality of life, safety, autonomy, and respect for human dignity.

Spain has made progress in establishing a national dementia strategy and providing caregiver support through leaflets, guidelines, and programs such as Cuidopía. However, unlike the best practices from the EU portal, Spain currently lacks structured, multidisciplinary programs. Early diagnosis pathways are not fully integrated, and systematic use of supportive digital tools or assistive technologies is absent. Existing initiatives are largely fragmented, focusing on advice and training rather than coordinated care across healthcare, social services, and family support. Incorporating elements from established best practices could strengthen national coverage, support early diagnosis, and expand access to digital tools for both patients and caregivers.

### 3.9 Ukraine

Ukraine has not yet developed a comprehensive national strategy on dementia; however, a number of policy and regulatory documents relating to the care of people with dementia have been adopted in recent years. These documents focus primarily on clinical care, as well as new policy initiatives aimed at raising awareness, coordinating healthcare and providing long-term support for people living with dementia and their caregivers.

The primary regulatory framework for dementia care is the updated National Guidelines and Standards of Medical Care “Dementia and Mild Cognitive Impairment” (2025) which defines diagnostic pathways, treatment approaches, and continuity of care for patients with dementia within the Ukrainian healthcare system.

The protocol defines clinical pathways which connect all medical facilities through different care levels while creating specific duties for general practitioners, neurologists, psychiatrists, psychologists, psychotherapists, hospice staff and all other professionals involved in dementia patient treatment. Primary care systems concentrate on identifying health problems during their initial stages while they treat conditions which can be changed through risk factor management that includes hypertension, diabetes, smoking, alcohol consumption and obesity, and they track mild cognitive impairment development and offer assistance to patients and their families. The healthcare system offers advanced diagnostic tests which include neuroimaging and neuropsychological evaluations together with their combination of drug-based and non-drug-based treatment methods. Palliative care provisions aim to maintain quality of life and dignity for people with advanced dementia while providing support for caregivers.

The evidence-based guidelines establish mandatory national standards which healthcare providers must follow to diagnose and treat dementia and mild cognitive impairment. The guidelines show that healthcare professionals from different specialties need to identify patients who have common dementia risk factors, such as low education level, hearing impairment, head injury, hypertension, obesity, diabetes, depression, social isolation, smoking, and environmental factors. The doctors must send patients who show signs of cognitive impairment to specialized medical professionals in neurology and psychiatry for assessment, which enables healthcare teams to start diagnosis and treatment earlier.

At the policy level, dementia care is also indirectly addressed through broader mental health reforms. The Action Plan for 2024–2026 for the implementation of the Concept for the Development of Mental Health Care in Ukraine until 2030 (33), approved by the Cabinet of Ministers of Ukraine in 2024, includes measures aimed at improving the quality of life of people with dementia and strengthening services for older adults with cognitive disorders.

Furthermore, a National Dementia Plan is currently under development (draft, 2025) by the Ministry of Health of Ukraine (34). The proposed plan establishes a national dementia care framework which will help raise public knowledge about dementia while decreasing its associated social stigma, improving access to medical and social support services, providing assistance to caregivers and enhancing government support for dementia care through policy development. The draft plan includes initiatives which will educate communities about dementia while making services more accessible to people with dementia through physical and cognitive accessibility enhancements.

In addition to formal healthcare policies, non-governmental actors play an important role in dementia care in Ukraine. One of the key organizations in this field is the “Nezabutni” (35) a national charitable foundation established in 2021, which supports people living with dementia and their families and promotes dementia-friendly communities.

The organization addresses multiple unmet needs through the provision of educational materials, practical guidance for caregivers, and awareness-raising initiatives aimed at reducing stigma and improving early recognition of dementia. It also delivers training programs and consulting support, strengthening informal care capacity in Ukraine.

The activities of “Nezabutni” reflect key elements of person-centered care, particularly in their emphasis on dignity, quality of life, and social inclusion. However, these initiatives remain largely confined to the civil society sector and are not yet systematically integrated into national health and social care systems.

Since 2019, Ukraine has been implementing the WHO-developed Mental Health Gap Action Program (mhGAP) Guidelines for the management of mental, neurological, and substance use disorders in non-specialized healthcare facilities. One of the sections of the guidelines is dedicated to dementia. Currently, training on mhGAP is mandatory for all family physicians who have a service contract with the National Health Service. Consequently, all family physicians are knowledgeable about the specifics of identifying and managing individuals with dementia.

In 2025, the mhGAP program began to be incorporated into the curricula of medical universities, which will help institutionalize the program.

Dementia care in Ukraine continues to follow a medical approach which lacks the implementation of integrated multidisciplinary person-centered treatment methods that exist in various European Union member states. Dementia support systems which include structured caregiver assistance programs, community-based service networks, digital dementia self-management and monitoring solutions require further development. Clinical guidelines establish procedures for early diagnosis yet healthcare facilities lack effective execution methods for cognitive rehabilitation programs and digital assistive technologies and structured training programs for both caregivers and healthcare professionals.

Ukraine has established a dementia diagnosis and treatment regulatory framework and launched national dementia strategy policy talks but the country needs to establish self-care support systems, caregiver training programs, community education initiatives and digital dementia care solutions which meet European best practices.

## 4 Discussion

This cross-country comparative analysis examined national guidelines and good practices on self-care, education and training, and digital tools for people with dementia and their caregivers in JADE Health partner countries. Across all countries, we observed considerable variation in maturity of dementia policies and services, but no setting implemented a model that comparably integrates structured self-care, systematic education, and scalable digital support (Table 1). Instead, existing measures tend to be fragmented, regionally limited, or focused on single components of care.

**TABLE 1:** Country comparison.

Country	Self-care	Education and training	Digital tools/robotics
Bulgaria	No structured self-care programs; access uneven beyond major centers.	Strong professional e-learning; limited systematic support for informal caregivers nationwide.	Telemedicine and GPS used, but no integrated self-management platforms or robotics.
Croatia	Regional programmes (PAM, SPAM) offer self-care support; no national framework.	Psychoeducational offers tied to regional projects; not standardized nationally.	Supportive digital tools and assistive robots currently absent.
Denmark	Policy promotes independence, but no explicit structured self-care model comparable to MCC.	Extensive staff training and guidelines; less systematic patient/caregiver education.	Uses apps and sensors (e.g., ReACT), yet no nationwide, structured digital or robotic solutions.
Germany	Guidelines touch lifestyle and psychosocial aspects; no dedicated self-care programme.	Strong professional standards; informal caregiver education remains fragmented.	Supportive digital tools and robotics not embedded in national guidance.
Latvia	Clinical pathways involve families but lack dedicated self-care interventions.	Focus on diagnosis; structured education for carers is missing.	No supportive digital tools or assistive robots identified.
Lithuania	Self-care largely supported informally through civil-society initiatives.	Some advocacy-driven training; no comprehensive national programme.	Institutional safety tech present; no systematic home-based digital tools or robotics.
Poland	Online caregiver guidelines support everyday self-care but are not programmatic.	Practical written guidance exists; multidisciplinary training structures lacking.	No structured digital or robotic solutions identified.
Spain	Leaflets and portals address self-care but remain fragmented.	Multiple materials for carers; no integrated, multidisciplinary education model.	Systematic implementation of digital tools and assistive technologies is lacking.
Ukraine	Clinical focus dominates; structured self-care support mostly absent.	NGO-based training exists but is not integrated nationally.	Digital self-management tools and robotics largely absent.

A first key finding is that self-care is scarcely established as a distinct and programmatic pillar of dementia care. Many countries mention lifestyle, psychosocial support, or caregiver relief in guidelines and policy documents, but concrete self-management structures, such as tailored coaching, structured group interventions, or routine follow-up embedded in care pathways, are rare. Where self-care support exists, it is often delivered through isolated initiatives (e.g., day hospital programs, municipal services) or civil-society activities, rather than as an integral part of national care models. This contrasts with the Memory and Cognition Consultation Program, which embeds self-care and caregiver support into a multi-professional longitudinal consultation framework.

A second important observation concerns education and training. Several countries have developed extensive training opportunities for health and social care professionals, including e-learning platforms, person-centered care curricula and practice-oriented guidelines. In contrast, structured, low-threshold education for informal caregivers and people with dementia themselves remains limited, unevenly distributed, and frequently project-based. Many caregiver resources are confined to brochures, websites or one-off courses that are not linked to formal pathways or quality assurance mechanisms. As a result, knowledge transfer and skill-building depend heavily on local initiatives and individual engagement, rather than on systematic, scalable programs.

The largest and most consistent gaps appear in the area of digital tools and robotics. Some countries have begun to implement telemedicine, safety technologies (e.g., GPS, motion sensors) or supportive apps that facilitate communication and orientation. However, these technologies are rarely integrated into coherent care pathways or combined with structured self-care and education components. Technologically advanced approaches, such as continuous tele-monitoring of cognitive function and lifestyle, multimodal digital training programmes, or robot-assisted assessment and interaction, as exemplified by Smart Aging Mindbrain and MARIO, are virtually absent from the national guidelines and good practices reviewed. This suggests that the main barriers lie less in technological feasibility and more in implementation strategies, reimbursement models, regulatory frameworks and ethical governance.

The analysis also highlights structural limitations in the current European infrastructure for identifying and scaling best practices. The EU Best Practice Portal provided a small number of relevant examples in the domains of self-care, education and digital tools, and several entries appeared to be dated in relation to rapid technological and service developments. It remains unclear whether this reflects a true scarcity of robust, evaluated best practices or rather gaps in documentation, submission and selection processes. Future work would benefit from combining portal searches more systematically with literature and gray-literature reviews, stakeholder mapping, and targeted calls for examples at national and regional level.

For JADE Health, particularly Work Package 9, these findings have several implications. First, pilots should explicitly address the three domains where gaps recur across all participating countries: programmatic self-care support, structured and sustained education for caregivers and people with dementia, and the integration of everyday digital solutions into person-centered care models. Rather than attempting to transfer complete reference models, it may be more feasible to adapt discrete elements, such as multi-professional consultation structures from the Memory and Cognition Consultation Program, tele-based cognitive and lifestyle interventions inspired by Smart Aging Mindbrain, or technology-supported assessment and social engagement elements from MARIO, to local systems and resources.

Second, the results point to the need for implementation strategies that go beyond piloting single projects. This includes aligning new care models with existing policies and financing mechanisms, defining clear roles and responsibilities across health and social sectors, and developing practical guidance for ethical and acceptable use of monitoring and assistive technologies. In parallel, strengthening national and regional capacities for co-design with people with dementia and caregivers will be essential to ensure that new self-care, education, and digital interventions are usable and acceptable in real-world contexts.

Third, the insights from this comparative analysis can help refine criteria for what should be considered a “best practice” in person-centered dementia care in Europe. Future best-practice frameworks could explicitly require multidimensional coverage of self-care, education and digital support, evidence of implementation beyond single pilot sites, and attention to equity and accessibility across urban and rural settings and vulnerable groups. Feeding these insights back into European repositories could improve the visibility and transferability of promising approaches and support more strategic selection of interventions for joint actions such as JADE Health.

### 4.1 Limitations

Within this analysis, several limitations need to be considered. First of all, we did not include measurable outcomes, and mainly limited our results to being descriptive and narrative. Therefore, one cannot assess the effectiveness of the identified national guidelines and good practices, and thus this analysis cannot give concrete evidence-based guidance. Another limitation of this paper is that despite our best efforts, we cannot be sure that we have found all national guidelines and good practices available in every country. This might be influenced by their visibility. Future research should mainly focus on effectiveness of national guidelines and practices and evaluate their overall impact on overall health, self-care, education, and digital support in dementia.

## 5 Conclusion

Overall, our findings underscore that many European countries have taken important steps in dementia policy, guideline development and local innovation, but that the non-pharmacological, person-centered domains of self-care, caregiver education and digital support remain underdeveloped and poorly integrated. Addressing these gaps offers a concrete opportunity for JADE Health to contribute to more sustainable, equitable and person-centered dementia care models across Europe.
